# How Is Intelligence Test Performance Associated with Creative Achievement? A Meta-Analysis

**DOI:** 10.3390/jintelligence9020028

**Published:** 2021-05-21

**Authors:** Maciej Karwowski, Marta Czerwonka, Ewa Wiśniewska, Boris Forthmann

**Affiliations:** 1 Institute of Psychology, University of Wrocław, 50-527 Wrocław, Poland; 2 Institute of Education, The Maria Grzegorzewska University, 02-355 Warsaw, Poland; mkwasnik@talent.edu.pl (M.C.); ewa.wisniewska@aps.edu.pl (E.W.); 3 Institute of Psychology in Education, University of Münster, 48149 Münster, Germany; boris.forthmann@wwu.com

**Keywords:** creative achievement, intelligence, meta-analysis

## Abstract

This paper presents a meta-analysis of the links between intelligence test scores and creative achievement. A three-level meta-analysis of 117 correlation coefficients from 30 studies found a correlation of *r* = .16 (95% *CI*: .12, .19), closely mirroring previous meta-analytic findings. The estimated effects were stronger for overall creative achievement and achievement in scientific domains than for correlations between intelligence scores and creative achievement in the arts and everyday creativity. No signs of publication bias were found. We discuss theoretical implications and provide recommendations for future studies.

## 1. Introduction

Both intelligence and creative thinking are critical for solving problems people face and dealing with uncertainty. Yet it is creative achievements—observable products or effectively applied ideas—that make the world around us better. Consider the recent COVID-19 crisis; thanks to scientists’ efforts, we are witnessing development of vaccines that help manage the virus threat (e.g., Waltz 2020). A confluence of multiple factors makes such creative achievements possible (Sternberg and Lubart 1996). There are robust cognitive (Plucker et al. 2019), personality (Feist 1998), and motivational (Csikszentmihalyi 1988) predictors of creativity but also temporal (Simonton 1977) and social (Lebuda 2016) circumstances that make creative achievement more or less likely.

In this paper, we focus on a cognitive factor that has been considered vital for creative achievement (see, e.g., Cox 1926). This factor is cognitive ability operationalized by intelligence test scores. As we discuss in more detail below, the test result is neither a perfect nor the only proxy of individuals’ cognitive skills. However, intelligence tests have been demonstrated to hold robust reliability and validity, including predictive validity, for a broad range of outcomes, including school results (Kuncel et al. 2005; Zaboski et al. 2018) and success in work (Hunter and Hunter 1984; Kuncel et al. 2004). Do they predict creative achievements as effectively? We rely on a meta-analysis to answer this question. First, however, we discuss the nuances and complexities of the main constructs we focus on.

The remainder of this paper is organized as follows. First, we define creative achievements by contextually embedding them within the Four-C framework of creativity (Kaufman and Beghetto 2009). Then, we focus more directly on and explain why a new meta-analysis is needed, given that a similar summary was published thirteen years ago (Kim 2008). Third, we dig deeper into the more complex relationship between intelligence and creative achievement, particularly discussing factors that might moderate the strength of estimated associations between these two constructs. As one of these potential moderators is the way creative achievement is measured, we discuss two recent instruments—the Creative Achievement Questionnaire (CAQ, Carson et al. 2005) and the Inventory of Creative Activities and Achievements (ICAA, Diedrich et al. 2018)—in a little bit more detail. Finally, we present the goals, methods, and results of the current investigation.

### 1.1. Creative Achievement in the Four-C Framework

Creative achievements differ in their magnitude and vary from quite mundane ones—take, for example, a new dish created to amaze the family—to the greatest scientific discoveries or art pieces, including those worth the Nobel prize (Lebuda and Karwowski, forthcoming). Therefore, creative achievements might be located on a continuum from little-c creativity to Pro-c, to Big-C creativity, while the most mundane, mini-c level is mainly associated with creative thinking and mental processes (Kaufman and Beghetto 2009). Little-c creativity denotes everyday, hobbyist activities (Ivcevic and Mayer 2009), usually conducted for the sake of solving a problem at hand (Zielińska et al. 2020). This level’s achievements are of personal rather than social importance, although sometimes they might be associated with favorable social reception as well. Indeed, it happens that everyday creativity results not only in activity but observable and recognized effects: internet memes, new ways of decorating the room, new meals, or jokes. Moreover, everyday or little-c creativity often serves as a prelude to more professional activities (Kaufman and Beghetto 2009).

Pro-c level denotes the creativity of someone who decided to engage in a specific domain (Sternberg 2002), spent some time, usually years, studying it (Kaufman and Kaufman 2007), and, often, uses creativity for a living. Many artists, scientists, and innovators (Florida 2019), but also managers or teachers, could be described as Pro-c creators. Thus, Pro-c is professionalized: creativity is a necessary aspect of one’s job. Given the changes in the job market, creativity is no longer required only from artists or scientists; as the World Economic Forum rightly recognizes, it forms a necessity of today’s world (see also Corazza 2016). However, the very fact that almost all jobs require creativity does not mean that all employees belong to the Pro-c creativity level. As Kaufman and Beghetto (2009, p. 5) explain: “Not all working professionals in creative fields will necessarily reach Pro-c (a professional actor, e.g., may make a good living on soap operas but may not necessarily be Pro-c level creative in his or her craft). […] Yet many ‘amateur’ artists are being creative at the Pro-c level, even if it is not their primary means of support.” Belonging to Pro-c means having observable, acclaimed achievements. These achievements do not necessarily revolutionize the domain at hand, yet they must be considered sufficiently original and valuable to be appreciated by the field.

Finally, Big-C creativity is the level that only very few geniuses attain. Needless to say, there is no Big-C creativity without products (Rhodes 1961) or artifacts (Glăveanu 2013) that push the domain into a new direction (Sternberg et al. 2002). Albert Einstein, Marie Curie, Thomas Edison, or Frida Kahlo, to name just a few, are examples of Big-C creativity.

In this paper and the meta-analysis we present below, we are particularly interested in the relationship between intelligence and creative achievement that is conceptually closest to little-c and Pro-c creativity levels. As Kaufman and Beghetto (2009) recognized, Big-C creativity is rare and hardly approachable in typical psychometric studies. Given that we aim at updating the previous meta-analytical synthesis, we rely on empirical studies that scrutinize the links between intelligence and creative achievement and—apart from specific examples of historiometric studies (e.g., Cox 1926)—they do not include Big-C samples.

### 1.2. Is a New Meta-Analysis Needed?

This paper aims to update the now 13-year-old meta-analysis of the links between intelligence and creative achievement (Kim 2008). Based on 17 studies with 5544 participants, this first synthesis presented a somewhat modest correlation between intelligence and creative achievement (*r* = .17, 95% CI: .14, .19). The studies included in Kim’s meta-analysis covered almost five decades (1958–2005). Here, our goal was to examine whether the links between intelligence and creativity reported in the last two decades have changed. Beyond the general importance for psychological science to update existing meta-analyses based on newly available cumulative evidence, we see three main reasons to scrutinize such a change.

First, new and robust measures of creative activities and achievements were introduced in the years past Kim’s meta-analysis, with two instruments being especially worth mentioning, namely the CAQ (Avitia and Kaufman 2014; Carson et al. 2005) and the ICAA (Diedrich et al. 2018). Both cover creative accomplishments across a broad range of different domains and quantify them from non-existent or relatively minor (observed in most cases) to professional-level creativity that received social acclaim (i.e., Pro-C creativity). A broader application of the CAQ and the ICAA opened new avenues for studying the role of cognitive (e.g., intelligence, see Carson et al. 2003), personality (e.g., Clark 2015; Gonzalez 2020; Sutu et al. 2019; Vellante et al. 2011), and motivational (e.g., Karwowski and Beghetto 2019) predictors of creative achievement. We discuss representative works that utilized the CAQ and the ICAA later on.

Second, yet related to the former, more consistent applications of standardized measures of creative achievements made the studies more comparable, thus reducing the risk of comparing apples and oranges often present in meta-analyses (Sharpe 1997). Indeed, previous studies often operationalized creative achievement in a hardly comparable manner. While some investigations used relatively objective measures, such as awards or publications (e.g., Feist and Barron 2003), others relied on open-ended questions and spontaneously developed lists of creative achievements that judges scored for their level of creativity (Plucker 1999). Although valid, such an approach often results in limited comparability.

Third, thanks to the increasing availability of online studies over the last two decades, larger investigations have been possible (e.g., McKay et al. 2017; Zabelina et al. 2020). Therefore, studies benefitted from increasing statistical power and stability of estimated correlations (Schönbrodt and Perugini 2013). In addition, intelligence tests became more available to be applied in online studies, with the most prevalent example of the International Cognitive Ability Resource (ICAR; Condon and Revelle 2014; Dworak et al. 2021). Taking all these factors into consideration, an update seems well justified. In this meta-analysis, we primarily focused on new studies, albeit we included some older investigations (e.g., pre-2005, and thus those included in Kim’s (2008) meta-analysis) for control and comparative purposes.

### 1.3. The Intelligence–Creative-Achievement Relationship and Its Moderators

What are the theoretical arguments to explore the link between intelligence and creative achievement? Classic studies considered intelligence as a necessary yet not sufficient condition of creative accomplishments (Cox 1926; Simonton 2013, see also Gough 1975, 1976; Lunneborg and Lunneborg 1968; McDermid 1965; Milgram and Milgram 1976). Although highly intelligent people are not always successful in creative domains (Shurkin 1992; but see also Park et al. 2007; Warne et al. 2020), above-average intelligence is often considered necessary to deal effectively with the requirements of creative domains (Karwowski et al. 2016; Plucker et al. 2019). Indeed, intelligence seems inevitable when it comes to learning of the “language” and rules of any creative domain. Thus, one line of theorizing and research on the intelligence–achievement links explored the possible linear-versus-non-linear character of relationships. Initially operationalized as a threshold hypothesis (Karwowski and Gralewski 2013; Weiss et al. 2020b) and more recently as a necessary-condition pattern (Karwowski et al. 2016), this approach searched for the non-linear relationship between intelligence and creative thinking (Karwowski et al. 2016) and achievement (Karwowski et al. 2017). However, such complex relationships cannot be reasonably treated in a meta-analysis (see Gerwig et al. 2021), and, hence, we focus here mainly on three moderators: domain of creative achievement, intelligence sub-factor, and creative achievement measure.

#### 1.3.1. The Domain of Creativity

Although creativity scholars disagree whether creative abilities are domain-general or domain-specific (Baer 2015; Plucker 1998), it goes without saying that achievement is domain-specific (Silvia et al. 2009b). Examples of multi-domain creators are scarce (Silvia et al. 2009b; Szen-Ziemiańska et al. 2017). Thus, an important question is whether the domain of creative endeavors might qualify the role intelligence plays. Some domains, be it science or engineering, are more cognitively demanding; others, such as social functioning or the arts, are probably less dependent on intelligence (Karwowski et al. 2017) but more on specific cognitive or social skills (Greengross and Miller 2009; Nemiro 1997). The reasons for a different role of cognitive ability might lie in both the domain’s complexity and early (self) selection to the domain. Indeed, choosing a professional career in science is more likely for students who do well in school (Kell et al. 2013) and those who are more intelligent (Benbow 1992), while considering a career in the arts is more likely for students who are particularly engaged in art-related activity out of school (Martin and Frenette 2017). Thus, the creativity domain is a natural candidate variable to moderate the relationships we are interested in. In domains that are more cognitively demanding, such as STEM fields, correlations are usually stronger (Karwowski et al. 2017; Kaufman et al. 2015), while they approach null in the case of arts or everyday creative behavior (see, e.g., Lunke and Meier 2016). Out-of-school creative activities (Jauk et al. 2014) are usually less dependent on intelligence, which obviously does not make intelligence not important here but rather denotes that its role might be less critical than in highly structured domains.

Kim’s (2008) meta-analysis analyzed domain as a moderator and—interestingly—obtained an average correlation estimate for science that was negligibly small (*r* = .06). The highest observed average correlation (*r* = .37) was observed for the links between intelligence and achievement in the domain of leadership, yet only four effects covered this domain. In addition, the previous meta-analysis focused on the domains of art, music, writing, science, leadership, and social skills. With the new tests, we are able to extend this to other domains, such as cooking or sports.

Creativity theories often emphasize the confluence of different personal and social factors explaining differences in creative activity and achievement (Jauk et al. 2014). As we already mentioned, personality (Feist and Barron 2003), creative confidence (Karwowski and Beghetto 2019), or deliberate practice (Ericsson 2014) are among factors that make creative accomplishments more likely. As Sternberg (2002) pointed out, creativity is a decision—it is easy to imagine that for many highly intelligent people, such a decision will not be particularly attractive to make. Therefore, although there are solid arguments for believing that intelligence matters for creative achievement, expecting that it will alone explain a large portion of the variability of creative achievements may be premature.

#### 1.3.2. The Theoretical Status of Intelligence and Creative Achievement

Another vital aspect to consider is the status of the measured phenomena when intelligence–creative achievement links are analyzed. By status, we mean the temporal dynamics of intelligence and creative achievement, their internal complexity, and how they are measured. Let us unpack this reasoning a little.

Although broad theories of intelligence were proposed in the past (Sternberg 1985) and more currently (Corazza and Lubart 2021; Sternberg et al. 2021), in virtually all studies we included in the meta-analysis presented below, intelligence is operationalized as a test’s or series of cognitive tests’ results. Most recent works that explore the links between creativity and intelligence are based on the Carroll–Horn–Cattell model (CHC, McGrew 2009), meaning that they analyze either the higher-level general factor (*g*, e.g., Karwowski et al. 2020) or links between creativity and medium-level factors, such as fluid (Gf, Miroshnik and Shcherbakova 2019) or crystallized (Gc, Sligh et al. 2005) intelligence. Both seem important for creativity—while *Gf* denotes domain-general ability to deal with novel problems, primarily by using deduction and induction (Nusbaum et al. 2014), *Gc* is more dependent on declarative and procedural knowledge that matters for domain-specific creativity. In the CHC model, mental operations important for creative thinking (e.g., originality of thinking or fluency) are categorized to the factor of general retrieval ability (*Gr*). Indeed, storing and effectively retrieving information in semantic memory serves as an important mechanism of creative associations. However, it must be remembered that recent theories strongly underlie the interaction between more spontaneous associative processes and controlled creative processes during creative thought (Beaty et al. 2015; Zabelina et al. 2019). Therefore, there are reasons to expect that creative achievement will benefit not only from general intelligence (*g*) but that it will be driven by domain-general mental effectiveness (*Gf*), domain-specific knowledge-based thinking (*Gc*), effective retrieval (*Gr*), or processing speed (Gs, see Forthmann et al. 2019) as well. While these different aspects of intelligence were already studied in relation to creative thinking, including a large recent meta-analytical summary (Gerwig et al. 2021), they were rather overlooked when it comes to creative achievement (but see Prabhakaran et al. 2014; Zabelina and Ganis 2018).

Kim’s meta-analysis included four different intelligence test families as a moderator (California Test of Mental Maturity, Lorge–Thorndike Intelligence Tests, Terman Concept Mastery Test, and others), yet not the specific aspects of intelligence. Among these tests, Lorge–Thorndike tests provided the highest correlation with creative achievement (*r* = .31, *k* = 17), and Terman tests (*k* = 32) resulted in a significantly weaker relationship (*r* = .18), while for the California Test of Mental Maturity (*k* = 41) and other measures (*k* = 4) the links were negligible (*r* = .07 and *r* = .03, respectively). As all these tests cover a broad range of abilities (see Carroll 1993), based on Kim’s summary alone, it is impossible to fully uncover which intelligence factor is stronger and which is correlated more weakly with creative achievement. This is one of the gaps we are going to fill in this meta-analysis by including the intelligence aspect as a potential moderator, differentiating the strength of the relationship between intelligence and creative achievement.

The second element to consider is the status and measurement of creative achievement. What counts as achievement varies depending on definition and operationalization. Indeed, sometimes the distinction between creative activity and behavior is not that clear. Consider a seminal measure of creative behavior—the Creative Behavior Inventory (Hocevar 1976; Silvia et al. 2021a). This instrument—initially developed as a 90-item measure, then shortened to a 28-item brief form (Dollinger 2003)—covers both creative activities and products (e.g., painted an original picture, made your own holiday decorations) but also recognition (e.g., received an award for an artistic accomplishment, receiving an award for making a craft). Similarly, the Biographical Inventory of Creative Behaviors (Batey 2007; Silvia et al. 2021b) covers such items as: “wrote a novel,” “published an article,” or “created a theory.” Whether these categories should be treated as indicating creative activity rather than achievement might seem disputable. The Creative Achievement Questionnaire (Carson et al. 2005) and the Inventory of Creative Activities and Achievements (Diedrich et al. 2018) are two recent examples of measures that explicitly focus on creative achievement and separate creative activity from creative achievement.

#### 1.3.3. CAQ and ICAA—A Closer Look

The CAQ covers ten domains of creativity: visual arts, music, dance, architecture, creative writing, humor, inventions, scientific discovery, music and film, and culinary arts. As the CAQ focuses on observable and socially recognized accomplishments, it serves as a primary measure of creative achievement—not only Pro-c but also, in rare cases, even Big-C creativity. The structure of the CAQ is similar across domains: in each domain, eight items describe the increasing level of creative achievement, starting from “no achievement/training in a particular domain” to awards received or works published. The results obtained are usually highly skewed, which resembles well the skewness of real-world creative achievement.

The CAQ is intensively used as a measure of creative achievement, along with several correlates and predictors of creativity. It has been robustly linked with divergent thinking (Carson et al. 2005), openness to experience (Silvia et al. 2009a), and creative activity (Beghetto et al. 2020) but not insight problem solving (Beaty et al. 2014; but see also Karwowski and Beghetto 2019). Regarding its links with intelligence, the results obtained to-date are quite heterogeneous. While some studies observed near-zero links (Kaufman et al. 2014), others reported small-to-moderate correlations of *r* = .14 (Carson et al. 2005; Harris et al. 2019) and *r* = .23 (Kéri 2011) to quite robust associations of β = .29 (Beaty et al. 2014) and *r* = .34 (Mar et al. 2006). A recent longitudinal study (Karwowski et al. 2017) that predicted CAQ scores in middle age (52 years old) by intelligence measured at age 11 and 13 showed negligible correlations with Raven matrices (*r* = .05, *p* = .047, N = 1594) and Wechsler Intelligence Scale for Children (*r* = .09, *p* = .077, N = 255). Still, the correlations were significant for achievement in science (*r* = .05 in Raven and *r* = .11 in WICS). Additionally, this longitudinal study did establish non-linear links between intelligence and creative achievement.

The ICAA is a relatively new measure (Diedrich et al. 2018) that is gaining popularity among creativity scholars. It covers creative activities and achievements across eight domains: literature, music, arts and crafts, creative cooking, sports, visual arts, performing arts, and science and engineering. The ICAA is a vital alternative for the CAQ, as it results in less skewed total scores and separates activities from achievements. Although robustly correlated (latent *r* = .68; Diedrich et al. 2018), the activity and achievement scales of the ICAA allow for a distinct measurement of two aspects of creativity. Diedrich and her colleagues demonstrated convincing validity of ICAA scales (e.g., art students scored higher than the representative sample on almost all domains except for cooking), and achievement in ICAA creative domains was robustly correlated with scores obtained in the CAQ. Regarding the links between ICAA achievement and intelligence, Diedrich et al. (2018) reported a relatively modest relationship, ranging from null in arts and crafts, cooking, and sports to *r* = .18 in science engineering. Aggregated correlations with ICAA sum scores varied between *r* = .09 in the case of numerical intelligence to *r* = .19 in figural intelligence. A study by Kellner and Benedek (2017) that utilized the ICAA had similar estimates with correlations estimated at *r* = .13 for *Gf*, *r* = .25 for *Gc*, and *r* = .23 for averaged score—a proxy of g.

Given that the ICAA was not yet available at the time when Kim conducted her meta-analysis, we assessed the measure of creative achievement as a moderator to explicitly assess whether Kim’s meta-analytical estimate of the correlation generalizes to these newer test concepts. We did not predict that these two scales will bring different estimates of intelligence–achievement links.

To summarize, the current overview of correlational studies between intelligence and creative achievement brings relatively modest effect sizes: ranging from *r* = .06 (Batey and Furnham 2008) to *r* = .11 (Kaufman et al. 2015), to *r* = .14 (Carson et al. 2005), and only very rarely exceeding *r* = .30, considered a large effect size for individual differences research (Gignac and Szodorai 2016). Although these associations increase (e.g., Beaty et al. 2014) when the measurement error is accounted for in latent variables models, the links are still lower than the latent correlations between intelligence and creative abilities, reported recently as being at about *r* = .40 (Benedek et al. 2012; Karwowski et al. 2020; Nusbaum and Silvia 2011; Weiss et al. 2020a).

Importantly, however, it must not be overlooked that the strength of the relationship between intelligence and creativity might depend on situational and contextual factors (e.g., see Corazza and Lubart 2021). The contextual conditions in which typical intelligence tests are applied are usually at odds with those in which the creative achievement emerges. Solving intelligence tests takes minutes; achieving something creative—years or decades. Therefore, even if our overview provides arguments to expect relatively moderate correlations between intelligence and creative achievement (e.g., around *r* = .20, Gignac and Szodorai 2016), the exact effect size obtained should be interpreted in light of the complexity of both creativity and intelligence, limitations of their measurement, and the temporal (i.e., “minutes versus decades”) discrepancy between these two constructs.

## 2. The Present Study

The current work updates Kim’s (2008) meta-analysis and includes relevant moderators that were not explored in this initial meta-analysis, namely, the intelligence facet, intelligence test’s modality (e.g., verbal, figural), creative achievement measurement, and differently categorized creativity domains. Additionally, as several studies reported clustered effects (i.e., there were many correlations from the same studies), we aimed to model clustering of the effect sizes and examine publication bias.

## 3. Materials and Methods

### 3.1. Literature Search and Initial Screening

To identify the primary literature relevant to this meta-analysis, we used a multimodal search strategy and performed searches in (i) electronic literature databases, (ii) academic journals, (iii) forward search of reference lists of articles, reviews, and meta-analyses (cross-reference check), and (iv) screening publication lists of scholars and contacting the authors of thematically relevant studies. The database search contained: Academic Search Ultimate, Education Source, ERIC, PsycINFO, PsycEXTRA, OpenDissertations, Google Scholar, and ResearchGate. The EBSCO and Science Direct search engines were used. Google Scholar and ResearchGate included publications in peer-reviewed academic journals, as well as gray literature (Adams et al. 2017; Karwowski 2020; Schmucker et al. 2017).

Whenever possible, we used Boolean search operators based on the following terms (keywords, abstracts, titles, and full text): intelligence* OR intellect OR IQ* OR cognitive abilities/ability*, AND creative achievement* OR creative accomplishment*, AND creative activities*. Research articles, book publications (searched by Google Books, Wiley Online Library), and dissertations containing these terms were initially selected and individually reviewed to find additional references. In the next step, apart from the search in databases, we hand-searched for articles in academic journals, reference and citation lists of reviews, and meta-analyses on the following topics: the relationship between intelligence and creativity, intelligence and divergent thinking, cognitive predictors of creative achievements, and cognitive predictors of creative activities.

When searching for studies, we introduced no restrictions concerning the time of publication. The meta-analysis covers a period that begins with early publications from the 1960s included in the previous meta-analysis (Kim 2008) and ends in March 2021. From the existing meta-analysis (Kim 2008), we could only reclaim the studies and effect sizes reported there to a limited extent because several publications were not available (unpublished reports or conference proceedings, books unavailable in authors’ universities’ libraries, etc.). Consequently, we included only those studies from the meta-analysis by Kim to which we had access, and we found some additional studies from that period (up to 2008). We then searched for more recent studies.

### 3.2. Screening and Eligibility Criteria

After the initial search, we applied specific criteria for screening the publications. Our meta-analysis focused on the relationship between intelligence and creative achievements; thus, we included correlational studies that reported this effect. First, we included accessible full-text or secondary resources that describe the results of quantitative studies published in English. We included only studies with sufficient statistical information to calculate the effect size, thus, data: (i) concerning effect size; (ii) concerning the size of the sample based on which a given effect was determined; (iii) concerning the measurement of intelligence; and (iv) concerning the measurement of creative achievements. We considered only the results of those studies in which participants’ general, fluid, or crystallized intelligence (one study also reported correlations between creative achievement and long-term storage and retrieval: Glr) was measured by standardized tests. We included studies in which non-clinical samples were involved. A few longitudinal studies available (e.g., Karwowski et al. 2017; Plucker 1999) were excluded for the sake of higher comparability.

Next, we excluded studies in which participants’ intelligence was defined and measured only in terms of executive functions, attentional flexibility, cognitive control, spatial skills, working memory, or school achievements assessed by SAT-M and SAT-V scores (e.g., Park et al. 2008). Studies reporting the relationship between intelligence and creative activities were also excluded^1^. Finally, we excluded studies involving gifted students (e.g., Makel et al. 2016; Robertson et al. 2010).

### 3.3. Coding Procedures

The first three authors independently extracted and coded the following information: sample size, effect size, intelligence test reliability, and creative achievement measurements. Moreover, we coded potential moderators that operated at study level or effect-size-level, such as (i) measures of intelligence and creative achievement; (ii) facet of intelligence measured (*g*, *Gf*, *Gc*, *Gr*); (iii) domain of creative achievement measured (e.g., everyday, arts, science, technological, overall); (iv) study procedure (group vs. individual; paper and pencil vs. computerized or online tests); (v) sample characteristics (participants’ average age, participant’s gender); (vi) location (country or continent in which study was conducted); and (vii) date of publication. The consistency was good: kappa ranged from 0.70 to 1, and inconsistencies were resolved during a discussion session until a perfect consensus was reached.

### 3.4. Study Selection

**Figure 1:**
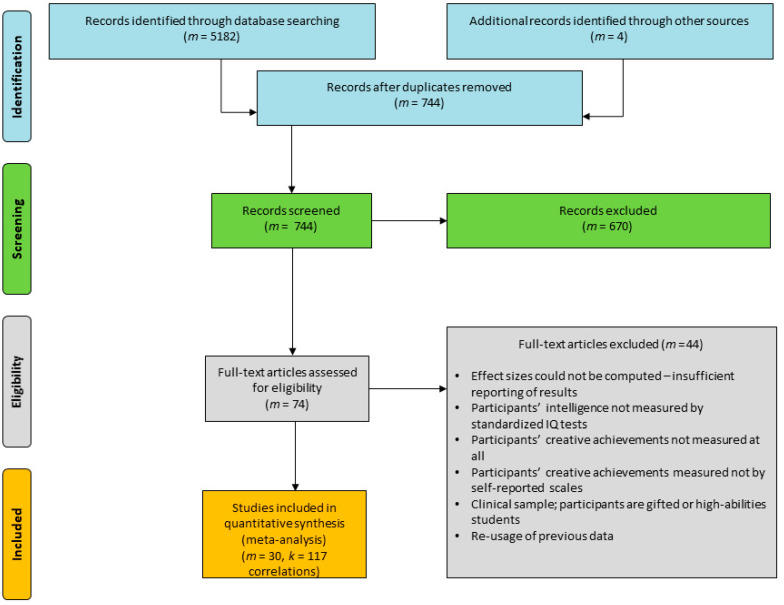
Flow diagram describing literature search and selection of eligible intelligence and creative achievements effect studies (adapted from the PRISMA Statement; Moher et al. 2009).

A flow diagram illustrates the process of study selection applied in our meta-analysis (see Figure 1). First, a total of *m* = 5182 studies, of which *m* = 5178 were found through queries in electronic databases using keywords, and *m* = 4 were found as a result of cross-reference literature analysis and hand-search. After removing duplicated records, two authors of this article read the titles, keywords, and abstracts of all the papers found (*m* = 744) using the inclusion criteria defined above. In the preliminary selection, we collected 74 studies. In the next stage, the selected sources were checked against the inclusion criteria. As a result, we excluded further 44 studies. The applied selection schema led us to the identification of 30 studies, which we included in the present meta-analysis.

### 3.5. Statistical Procedure

Given that most coefficients were clustered within studies, we relied on a three-level meta-analysis (Konstantopoulos 2011). This statistical method splits the variance into sampling variance and two sources of true variance: between-study variance, accounting for the variability between the studies, and within-study variance, accounting for the variability within the different studies (i.e., between effect sizes within each study). As a result, estimates of standard errors are unbiased and more robust. The true variance was estimated using the restricted maximum likelihood estimator (REML) to avoid bias (Viechtbauer 2005). Given that most of our moderator variables were categorical, meta-regression was computed with mean cell coding to facilitate the results’ interpretations. Subsequently, a series of linear hypothesis tests were applied to test the differences of the estimated effects for the various combinations of moderator levels. All analyses were conducted in R (R Core Team 2016), using the metafor (Viechtbauer 2010) and metaviz (Kossmeier et al. 2020) packages. 

## 4. Results

The data provided 117 coefficients from 30 studies (*N* = 21,748)^2^. Mean sample age ranged from 17 to 42 years, which implies that participants were young overall (*M* = 27.11, *SD* = 7.15). Most studies were from the USA (*m* = 19; the other countries included Austria, Poland, Canada, Hungary, Israel, Sweden, Switzerland, and the UK).

### 4.1. Overall Effect

**Figure 2:**
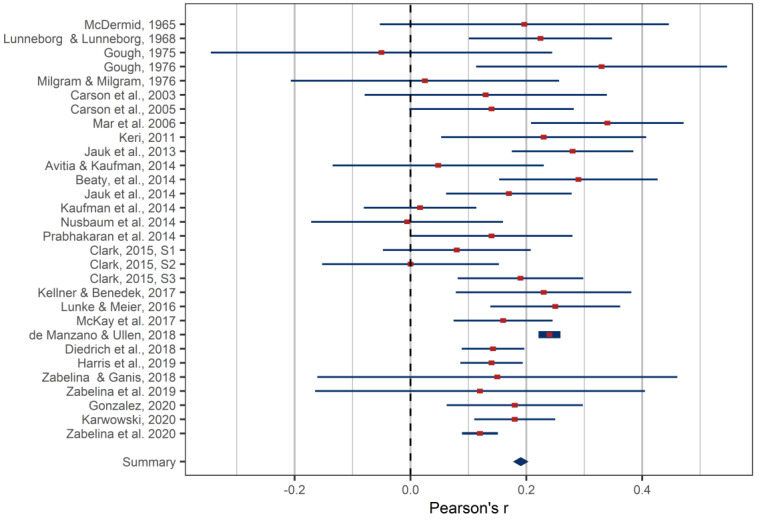
Forest plot with the aggregated effect size (Person’s r) of the correlation between intelligence and creative achievement for all studies included in the meta-analysis. Please note that as all analyses reported in this article used multilevel models, there are slight differences between the effects reported in Figure 1 and the effects estimated in the multilevel meta-analysis, including the overall effect, which is drawn at *r* = .18 (aggregated across studies) rather than *r* = .16 obtained in the multilevel meta-analysis.

The estimated overall correlation between creative achievement and intelligence was *r* = .16, 95% CI: .12, .19 (*m* = 30, *k* = 117) (Figure 2). Level-2, namely within-studies (between-effects), and Level-3 (between-studies) variance was statistically significant (τ_Level-2_ = .005, *SE* = .001, *p* = .002 and τ_Level-3_ = .005, *SE* = .002, *p* = .02).^3^ Overall heterogeneity was significant: *Q*(*df* = 116) = 498.48, *p* < .001, with *I*^2^ for Level-2 being estimated at 37% and *I*^2^ for Level-3 at 38%.

As our meta-analysis covered some large studies (e.g., de Manzano and Ullén 2018, *N* = 9537), which might have influenced the overall effect heavily, we tested for the sensitivity of the estimated overall effect size for outlying and large studies. We conducted the leave-one-out analysis, and thus we performed a series of new meta-analyses, each time excluding one effect. The results are presented in Supplementary Material (SM), Table S1. The effects ranged from *r* = .159 to *r* = .173, showing that the overall effect we reported was fairly robust.

### 4.2. Moderator Analysis

We included three continuous moderators (study year, percentage of females, average age of participants), as well as five categorical ones (study before or after Kim’s meta-analysis, creativity domain, creative achievement measure, intelligence facet, and the modality of intelligence test). Continuous moderators were introduced for control purposes; namely, we did not hypothesize any specific effects regarding study year or sex/age composition of the sample for our estimates.

#### 4.2.1. Study Characteristics: Year and Sample Composition

We started with a multilevel model that regressed the effect size on the study’s year (grand mean centered), percentage of female participants, and average age of participants in the study (grand mean centered). None of these effects yielded statistically significant differences in the effects we obtained: year (*b* = −0.002, *SE* = 0.001, *p* = .13), percent of female participants (*b* = 0.07, *SE* = 0.05, *p* = .13), or average age of participants (*b* = 0.003, *SE* = 0.002, *p* = .21). When we compared the effects obtained in studies before Kim’s meta-analysis (*k* = 24, *m* = 7, total *N* = 812) and after this meta-analysis (*k* = 93, *m* = 23, total *N* = 20,936), we did not observe statistically significant differences either (*Q_M_*(1) = 0.24, *p* = .62): *r*_before_ = .18, 95% *CI*: .10, .26, *p* < .001, *r*_after_ = .16, 95% *CI*: .12, .19, *p* < .001.

#### 4.2.2. Creativity Domain

We coded the domain of creativity in the following way: “total achievement” denoted the overall, aggregated score across different domains as available in most popular instruments (e.g., CAQ, ICAA). Writing, visual arts, music, dance, and theater were classified as the arts while architecture, engineering and scientific achievements as science. Finally, achievement in sport, cooking, humor, or games was classified as everyday creativity.

The creativity domain significantly moderated the effect size we obtained, *Q_M_*(3) = 221.67, *p* < .001 (see Table 1). When creative achievement was operationalized as domain-general total score, the correlation (*r* = .16) was found to be significantly stronger than in the case of the creative achievement in the arts (*r* = .09, *p* < .001, contrast = 0.08, *SE* = 0.009, *p* < .001) and everyday creativity (*r* = .06, *p* < .001, contrast = 0.10, *SE* = 0.01, *p* < .001) yet weaker than in the case of creativity in science (*r* = .19, *p* <.001, contrast = −0.02, *SE* = 0.009, *p* = .013). The correlation between intelligence and creative achievement in science was also stronger than for the links between intelligence and creativity in the arts and everyday domains (both *p*s < .001).

#### 4.2.3. Creative Achievement Measure

Among studies included in the current synthesis, twenty were based on the CAQ, and four utilized the ICAA. Six other creative achievement measures were used (Table 2). The overall omnibus effect was not significant, *Q_M_*(2) = 2.76, *p* = .25, although studies that utilized the CAQ tended to provide slightly weaker effect size. Planned contrast did not demonstrate any statistically significant differences (all *p*s > .05).

#### 4.2.4. Intelligence Facet and Test Modality

We observed a statistically significant moderator effect in the case of the intelligence facet (see upper panel of Table 3: *Q_M_*(3) = 9.30, *p* = .026) but no differences between different modalities of intelligence tests: verbal, figural, or both: *Q_M_*(2) = 1.54, *p* = .46. As illustrated in Table 3, studies that measured *Gc* provided a lower overall intelligence–creative-achievement correlation than was observed in the case of *g*, *Gf*, and *Gr* (one study only). The correlation between creative achievement, *g*, *Gf*, and *Gr* was significantly stronger than the link between creative achievement and *Gc* (*p* = .043, *p* = .048, and *p* = .036, respectively). The contrast between achievement–*g* links and achievement–*Gr* correlations was not significant (*p* = .52), similarly to the differences between the achievement–*Gf* relationship and the achievement–*Gr* link (*p* = .55).

### 4.3. Publication Bias

**Figure 3:**
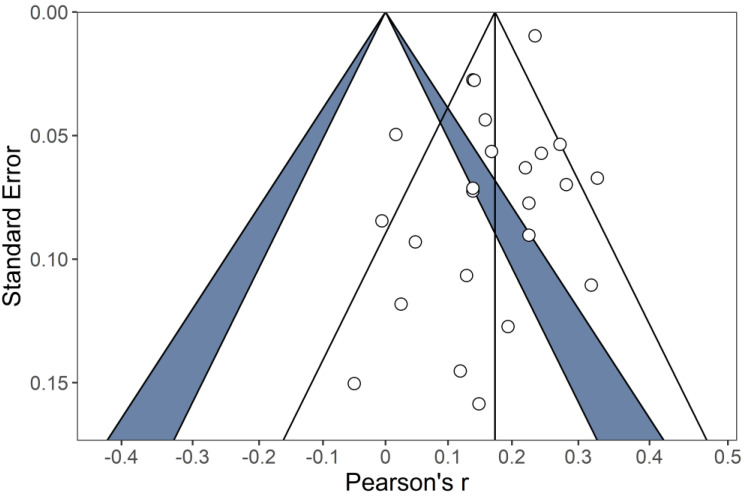
Funnel plot to test publication bias. Dots are studies. Only published studies are included (*m* = 24).

To check for possible publication bias, we consulted a funnel plot (Duval and Tweedie 2000) that included published studies only (see Figure 3). The distribution for effect sizes was symmetric, which was confirmed by the non-significant Egger test (*z* = −0.89, *p* = .37). Additionally, we compared the results obtained in published and unpublished studies. As no significant differences were found, QM(1) = 0.63, *p* = .43, with effects in unpublished studies being estimated at *r* = .13 (95% *CI*: .06, .21) and in published studies at *r* = .17 (95% *CI*: .13, .20), we conclude that it is unlikely that publication bias influenced our estimates.

## 5. Discussion

This meta-analysis extends and updates a summary published thirteen years ago (Kim 2008) that included studies conducted before 2005, mainly from the period of 1960–1980. By having thirty, mostly recent studies and more than a hundred correlations between intelligence and creative achievement, we almost precisely replicated the previous point estimate of *r* = .16^4^. This correlation was unlikely to be influenced by outlying effects or publication bias. Therefore, the first conclusion our meta-analysis provides is that the relationship between intelligence and creative achievement, albeit statistically significant, is small-to-moderate in terms of the effect size (Gignac and Szodorai 2016). We were unable to correct this effect for unreliability, as only nine studies reported reliability for both creative achievement and intelligence. However, we note that the average reliability of intelligence scores and creative achievement scores in our database was the same: α = .75. Taking this value as a proxy for reliability in the rest of the studies, the unreliability-corrected effect size grows to *r* = .21 (which is considered typical for individual differences research; see, e.g., Gignac and Szodorai 2016). We emphasize, however, that this point estimate should be read in light of the nuances we discussed in the introduction. First, intelligence tests’ results are only proxies (even if good proxies) for intelligent behavior. Second, there is a large difference between the status of intelligence and creative achievement. This difference is conceptual: intelligence tests focus on individual characteristics, and creative achievement is the effect of interaction between cognition, motivation, personality, and social reception. This difference is also temporal—taking tests takes minutes, while creative achievements take time. Keeping this in mind might change the interpretation of the overall effect we obtained. While we would not call it strong, we restrain from considering it trivial either.^5^

A meta-analytical summary of bivariate correlations is unable to provide insight into the possibly subtler patterns of relationships: be it non-linear links (a threshold theory, see, e.g., Jauk et al. 2013; Karwowski et al. 2017) or testing whether intelligence could be considered a necessary-yet-not-sufficient-condition for creativity (e.g., Karwowski et al. 2016). At least some datasets included in this synthesis suggest such a possibility, yet full access to raw data is necessary in order to test it formally. As Karwowski et al. (2016) demonstrated, the NCA could occur even if there is virtually no correlation between variables. Thus, apart from the average correlation obtained, we invite future researchers to follow the open science practices and share their raw data to test more nuanced links in the future.

The relationship we observed was independent of most analyzed studies’ characteristics: the percentage of male and female participants, their average age, or studies’ year. Indeed, when compared to the effects obtained in studies conducted before the previous meta-analysis (Kim 2008) that covered studies published before 2005, we did not observe significant differences in terms of the effect size. This similarity is serving the robustness of overall findings.

Two of the moderators included in our analyses significantly modified the correlation we obtained. First, the correlation was significantly stronger when the links between intelligence, total creative achievement, and creative achievement in science were compared to those obtained for the arts and everyday creativity. In the case of total creative achievement, namely the total score in the CAQ or the ICAA, the stronger correlation seems to be caused by higher variability of the longer scales and their higher reliability. It is also possible that the reason lies in polymathy. Polymathy (i.e., learning and expertise in multiple domains; Araki 2018) might be reflected in total creative achievement scores (i.e., higher scores imply achievements in multiple domains). Intelligence is considered an antecedent of polymathic knowledge needed to achieve accomplishments in various domains (Araki 2018). Expertise research that focuses on single domains instead of multiple domains has proposed a similar developmental connection between intelligence and required domain knowledge and skills (Deary et al. 2007). However, when domain expertise (be it polymathic or not) has sufficiently matured, intelligence’s predictive power is likely to collapse (Krampe and Charness 2018). Hence, a relatively small correlation between intelligence and creative achievement—again, be it polymathic or not—should not be strong when both constructs are concurrently measured (mainly when the amount of domain learning is not controlled).

In the case of comparisons between domains, however, the differences were more substantial. Indeed, art and science were found to be driven by different profiles of personality (Feist 1998; Kaufman et al. 2015) and cognitive factors (e.g., Kaufman et al. 2015; Karwowski et al. 2017). As highlighted in the introduction, more cognitively demanding creative domains, such as science, might be more intelligence-dependent than everyday behavior. That being said, across all domains we analyzed (art, science, and everyday creativity), the correlations were positive and statistically significant, even if they differed. Interestingly, our estimates of intelligence–achievement links in the case of science (*r* = .19) were visibly higher than the links reported by Kim (2008, *r* = .06).

We also observed that the CAQ (Carson et al. 2005)—the most often applied measure of creative achievement nowadays—resulted in somewhat lower correlations with intelligence (average *r* = .14) as compared to the ICAA (Diedrich et al. 2018, average *r* = .20) and other measures (*r* = .20), yet these differences were not statistically significant. While this slight difference could lead to perceiving the CAQ as too difficult, suffering from a severe floor effect, and, therefore, a measure that artificially lowers the relationship between intelligence and creative achievement, such a conclusion seems premature in light of plausible alternative explanations. The main reason why the CAQ resulted in lower estimates stems from the fact that it was the CAQ that provided most of the domain-level estimates. Due to the small number of effects, the ICAA and other measures were analyzed using total scores rather than domain scores. Given that domain scales of the CAQ are difficult (low scores prevail), relatively short, and not always very reliable, that might influence the overall effect obtained. We suggest that future researchers develop longer scales covering creative achievement across different domains that would be more robust to the current issues with reliability and floor effects (see also Silvia et al. 2012).

Our analyses demonstrated that the links between intelligence and creative achievement tended to be slightly weaker (*r* = .11) when *G*c was measured as compared to correlations with *g* (usually overall scores in intelligence tests), *Gf*, and *Gr* (only one study). Given that the remaining correlations were quite similar (*r*s between .17 and .20), this outlying effect is intriguing. Still, however, only seven studies included in this meta-analysis had *Gc* measures, and thus future studies are needed to provide a more solid test of the role of the intelligence facet for creative achievements, especially across domains. Given that a recent comprehensive meta-analysis (Gerwig et al. 2021) of the links between intelligence and divergent thinking did not reveal such a difference for the relationships between divergent thinking and *Gc* (*r* = .28), as compared to the relationship between divergent thinking and *Gf* (*r* = .23), it seems worthwhile to recommend that future research uses a more comprehensive measurement of intelligence, likely based on the Cattell–Horn–Carroll approach (McGrew 2009) as well as a more fine-grained, domain-specific measurement of creative achievement.

## 6. Limitations

The results of this meta-analysis should be read in light of some limitations. There are at least four such aspects that should be addressed in future studies. The first is that we could not provide measurement error-corrected effects, as most studies did not report reliabilities for creative achievement measures. As the CAQ has been the primary measure of creative achievement in the last decade, and the items within CAQ scales are not independent, calculating alpha seems psychometrically doubtful (see, e.g., Silvia et al. 2012). Still, future meta-analysts would benefit from the possibility of correcting the estimates for imperfect reliabilities. Therefore, alternatives to internal consistency measures should be strongly considered: be they reliability scores obtained from Item Response Theory or test–retest correlations.

The second limitation is the lack of possibility to analyze the correlations at the level of specific creative domains. We decided to merge different domains into three broad groups—art, science, and everyday creativity—yet such a decision may raise apparent doubts. More studies using established measures, such as the CAQ (Carson et al. 2005) and the ICAA (Diedrich et al. 2018), and reporting effects at the total achievement score level and each of the domains included are needed. We also invite fellow researchers to use our raw data and re-categorize the domains.

Third, the age of participants included in the studies was relatively low, with an average of 27 years. Creative achievement requires time and training, and thus there is a risk that these samples suffered from range restriction and therefore underestimated the relationships between intelligence and creative achievement. Moreover, most studies came from the so-called WEIRD (western, educated, industrialized, rich, and democratic) countries—a factor that might further limit our findings’ generalizability.

Fourth and final, in this meta-analysis, we were unable to examine whether the links between intelligence and creative achievement are non-linear or whether they follow a pattern suggesting that intelligence is a necessary yet not sufficient condition for creativity. The threshold hypothesis still raises the interest of creativity researchers (see, e.g., Jauk et al. 2013; Karwowski and Gralewski 2013), and there is a theoretically and empirically (Karwowski et al. 2017) convincing possibility that intelligence serves as a necessary condition for creative accomplishments. As raw data are needed to provide a test for such hypotheses, we hope that the growing acceptance for open science practices will make it possible to examine these links shortly.

## 7. Conclusions

This meta-analysis replicated previous links between intelligence and creative achievement. However, it provided arguments to consider intelligence’s role as more critical in creative achievement in science than in art and everyday creativity. The overall moderate correlations support complex theoretical models of creative behavior and achievement that incorporate cognitive potential (intelligence, divergent thinking) as well as particular personality and motivational traits (see, e.g., Karwowski and Beghetto 2019).

## Figures and Tables

**Table 1:** Effect size of the link between creative achievement and intelligence as a function of the domain of creative achievement.

		95%-CI					
Effects	Estimate	LB	UB	*P*	*k*	*m*	*N*
Total Achievement	.16	.13	.19	<.001	31	22	19,983
Achievement—Arts	.09	.06	.12	<.001	69	13	15,317
Achievement—Science	.19	.16	.22	<.001	56	16	15,444
Achievement—Everyday	.06	.03	.10	<.001	26	6	3482

LB = Lower Bound; UB = Upper Bound; *k* = number of coefficients; *m* = number of studies; *N* = total sample size.

**Table 2:** Effect size of the link between creative achievement and intelligence as a function of the measure of creative achievement.

		95%-CI					
Effects	Estimate	LB	UB	*p*	*k*	*m*	*N*
CAQ	.14	.10	.17	<.001	86	20	18,849
ICAA	.20	.11	.29	<.001	7	4	2002
Other	.20	.12	.28	<.001	23	6	752

LB = Lower Bound; UB = Upper Bound; *k* = number of coefficients; *m* = number of studies; *N* = total sample size.

**Table 3:** Effect size of the link between creative achievement and intelligence as a function of intelligence facet and intelligence test modality.

		95%-CI					
Effects	Estimate	LB	UB	*p*	*k*	*m*	*N*
*g*	.17	.13	.21	<.001	36	20	9600
*Gf*	.17	.12	.22	<.001	46	7	12,761
*Gc*	.11	.06	.17	<.001	25	7	2169
*Gr*	.20	.11	.29	<.001	10	1	116
							
Verbal	.17	.13	.21	<.001	43	10	2527
Figural	.17	.13	.22	<.001	27	7	12,761
Both	.15	.11	.18	<.001	47	20	9801

LB = Lower Bound; UB = Upper Bound; *k* = number of coefficients; *m* = number of studies; *N* = total sample size.

## Data Availability

Datasets and scripts used for the current meta-analysis are available in OSF: https://osf.io/6saej/?view_only=3916d2f5c45e4405a96ca884be2b26d5.

## Notes

1 Notably, studies with a focus on creative activities may have assessed creative achievements as another variable. In some rare cases, we expected to find such studies only when the search term also includes “creative activities” (see Section 3.1).

2 Nine additional studies focused on the relationships between intelligence and creative activity rather than creative achievement. We decided not to include these studies, but we did note that the effect size for the activity-intelligence links observed in these studies was weaker: *r* = .09, 95% *CI*: .05, .13, *p* < .001 than reported.

3 When estimating this overall effect, only the total effect was included in studies that reported both the correlation between the total (aggregated) creative achievement and intelligence and correlations between intelligence and specific creative domains. Three reasons drove this decision. First, the total score is more reliable than the scores obtained in shorter scales of specific domains. Second, it is less likely that the total score suffers from restriction of range and floor effects—a common problem of domain scores. Third and final, in such studies, we omitted domain-based correlations to avoid including them twice (as already covered by the total score). The domain-level correlations were used to analyze the role of the domain moderator, presented later on.

4 It is fascinating to note that our point estimate of *r* = .16 (95% *CI*: .12, .19) perfectly overlaps with the correlation between IQ estimates and eminence in a sample of geniuses in Cox (1926) study. More specifically, after controlling for IQ scores reliability, Cox obtained a correlation of *r* = .16, with a 95% *CI*: .12, .20 (Cox 1926, p. 55). We are grateful to Dean Keith Simonton for bringing this to our attention.

5 We appreciate the thoughtful comment of the anonymous reviewer who pushed us to re-think this issue.

## Supplementary Materials

The following are available online at https://www.mdpi.com/article/10.3390/jintelligence9020028/s1, Table S1: The leave-one-out meta-analysis results.
