# Supplementary material for: How Is Intelligence Test Performance Associated with Creative Achievement? A Meta-Analysis
Source: J Intell. 2021 May 21;9(2):28. doi: 10.3390/jintelligence9020028 (PMC8162535; doi:10.3390/jintelligence9020028)
Supplement: Supplementary file 1 [file jintelligence-09-00028-s001.zip › jintelligence-1003565-supplementary.pdf]

Table S1. The leave-one-out meta-analysis results.

Study	estimate	se	95% CI LB	95% CI UB	pval	Q	Qp	tau2	I2	H2
1	0,16	0,02	0,13	0,19	0,00	119,91	0,00	0,00	72,12	3,59
2	0,16	0,02	0,13	0,19	0,00	118,93	0,00	0,00	71,24	3,48
3	0,16	0,02	0,13	0,19	0,00	117,03	0,00	0,00	69,57	3,29
4	0,16	0,02	0,13	0,19	0,00	119,84	0,00	0,00	72,09	3,58
5	0,16	0,02	0,13	0,20	0,00	117,49	0,00	0,00	71,84	3,55
6	0,16	0,02	0,13	0,19	0,00	120,00	0,00	0,00	72,15	3,59
7	0,16	0,02	0,13	0,19	0,00	120,06	0,00	0,00	72,10	3,58
8	0,16	0,02	0,13	0,20	0,00	118,10	0,00	0,00	71,60	3,52
9	0,16	0,02	0,13	0,19	0,00	117,96	0,00	0,00	70,51	3,39
10	0,17	0,02	0,14	0,20	0,00	108,62	0,00	0,00	66,76	3,01
11	0,16	0,02	0,13	0,20	0,00	117,82	0,00	0,00	71,91	3,56
12	0,16	0,02	0,13	0,19	0,00	120,00	0,00	0,00	72,28	3,61
13	0,16	0,02	0,13	0,19	0,00	119,88	0,00	0,00	72,56	3,64
14	0,16	0,02	0,13	0,20	0,00	117,82	0,00	0,00	71,47	3,51
15	0,16	0,02	0,13	0,19	0,00	118,52	0,00	0,00	71,27	3,48
16	0,16	0,02	0,13	0,19	0,00	119,85	0,00	0,00	72,10	3,58
17	0,16	0,02	0,13	0,19	0,00	120,25	0,00	0,00	72,18	3,59
18	0,16	0,02	0,13	0,20	0,00	118,43	0,00	0,00	71,66	3,53
19	0,17	0,02	0,14	0,20	0,00	101,45	0,00	0,00	67,26	3,05
20	0,16	0,02	0,13	0,19	0,00	120,21	0,00	0,00	72,08	3,58
21	0,16	0,02	0,13	0,19	0,00	119,87	0,00	0,00	72,57	3,65
22	0,16	0,02	0,12	0,19	0,00	61,13	0,00	0,00	59,40	2,46
23	0,16	0,02	0,13	0,20	0,00	117,66	0,00	0,00	71,65	3,53
24	0,17	0,02	0,13	0,20	0,00	114,61	0,00	0,00	69,87	3,32
25	0,16	0,02	0,13	0,19	0,00	120,25	0,00	0,00	72,61	3,65
26	0,16	0,02	0,13	0,19	0,00	114,86	0,00	0,00	68,00	3,13
27	0,17	0,02	0,13	0,20	0,00	115,17	0,00	0,00	70,18	3,35
28	0,16	0,02	0,13	0,20	0,00	100,67	0,00	0,00	67,63	3,09
29	0,16	0,02	0,13	0,19	0,00	119,94	0,00	0,00	72,76	3,67
30	0,16	0,02	0,13	0,19	0,00	120,24	0,00	0,00	72,49	3,63
31	0,16	0,02	0,13	0,19	0,00	120,25	0,00	0,00	72,68	3,66
